# Conserved motifs in nuclear genes encoding predicted mitochondrial proteins in *Trypanosoma cruzi*

**DOI:** 10.1371/journal.pone.0215160

**Published:** 2019-04-09

**Authors:** Lorena Becco, Pablo Smircich, Beatriz Garat

**Affiliations:** 1 Laboratorio de Interacciones Moleculares, Facultad de Ciencias, Universidad de la República, Montevideo, Uruguay; 2 Departamento de Genómica, Instituto de Investigaciones Biológicas Clemente Estable, Ministerio de Educación y Cultura, Montevideo, Uruguay; Instituto Butantan, BRAZIL

## Abstract

*Trypanosoma cruzi*, the protozoan parasite that causes Chagas’ disease, exhibits peculiar biological features. Among them, the presence of a unique mitochondrion is remarkable. Even though the mitochondrial DNA constitutes up to 25% of total cellular DNA, the structure and functionality of the mitochondrion are dependent on the expression of the nuclear genome. As in other eukaryotes, specific peptide signals have been proposed to drive the mitochondrial localization of a subset of trypanosomatid proteins. However, there are mitochondrial proteins encoded in the nuclear genome that lack of a peptide signal. In other eukaryotes, alternative protein targeting to subcellular organelles via mRNA localization has also been recognized and specific mRNA localization towards the mitochondria has been described. With the aim of seeking for mitochondrial localization signals in *T*. *cruzi*, we developed a strategy to build a comprehensive database of nuclear genes encoding predicted mitochondrial proteins (MiNT) in the TriTryps (*T*. *cruzi*, *T*. *brucei* and *L*. *major)*. We found that approximately 15% of their nuclear genome encodes mitochondrial products. In *T*. *cruzi* the MiNT database reaches 1438 genes and a conserved peptide signal, M(L/F) R (R/S) SS, named TryM-TaPe is found in 60% of these genes, suggesting that the canonical mRNA guidance mechanism is present. In addition, the search for compositional signals in the transcripts of *T*. *cruzi* MiNT genes produce a list, being worth to note a conserved non-translated element represented by the consensus sequence DARRVSG. Taking into account its reported interaction with the *T*. *brucei* TRRM3 protein which is enriched in the mitochondrial membrane fraction, we here suggest a putative zip code role for this element. Globally, here we provide an inventory of the mitochondrial proteins in *T*. *cruzi* and give evidence for the existence of both peptide and mRNA signals specific to nuclear encoded mitochondrial proteins.

## Introduction

*Trypanosoma cruzi* (Kinetoplastidae, Trypanosomatidae) is the protozoan parasite that causes Chagas’ disease, also known as American trypanosomiasis [1]. This disease affects 6 to 7 millions of people, mostly from poor rural regions of 21 countries of Central and South America, where the vector responsible for the transmission to humans, diverse species of the Reduvidae, is found [2]. Nevertheless, since the parasite can also be transmitted by contaminated food, congenitally from mother to child and through contaminated blood or organ donations, disease has spread out world-wide.

Kinetoplastids are characterized by the presence of a dense structure of DNA and proteins at their unique mitochondrion, the kinetoplast. Therein two types of circular and concatenated DNA are assembled: the maxi and minicircles. The maxicircles (20–37 kb) are functionally equivalent to the mitochondrial DNA of other eukaryotes, and contain the genes encoding rRNAs (12S rRNA and 9S rRNA) and a reduced number of proteins (ND1; ND3;ND4; ND5; ND8; ND9; MURF1; MURF2; MURF5; COI; COII; COIII; Cyb; ATPase6; CR3; CR4; RPS12) [3]. Their transcripts need to be extensively edited (uridine addition/deletion) to solve features such as discontinuous open reading frames (ORFs), absence of essential elements for translation, i.e. initiation codons, or extensive modification to generate ORFs [4–10]. The minicircles (0.5–2.8 kb) contain the huge repository of sequences encoding the guide RNAs (gRNA) which drive the editing process [8,11,12], albeit a few gRNAs are encoded in the maxicircles [13]. All the other mitochondrial proteins are encoded in the nuclear parasite genome. So, in spite of the fact that the mitochondrial DNA constitutes up to 25% of total cellular DNA, the structure and functionality of the mitochondrion (oxidative ATP synthesis, redox balance [14], among others) are absolutely dependent on the expression of the nuclear genome. Proteomic analyses in *T*. *brucei* have enabled the identification of 1065 mitochondrial proteins from which only 18 are encoded in the mitochondrial genome [15].

In eukaryotes, the mitochondrial localization of proteins encoded in the nuclear genome is paradigmatically achieved through specific peptide signal [16]. This is also the case for trypanosomatids [17,18]. However, there are mitochondrial proteins encoded in the nuclear genome that do not present peptide signal. Afterwards, protein subcellular localization facilitated through mobilization of mRNA towards organelle surrounds has been recognized in eukaryotes [19] and later in *T*. *cruzi* [20]. Particularly, in eukaryotes but not yet in *T*. *cruzi*, specific mRNA localization towards the mitochondria has been described [21,22].

In order to study the existence of putative mitochondrial localization signals in *T*. *cruzi*, a database of nuclear genes encoding predicted mitochondrial proteins was built (MiNT). We could establish that *T*. *cruzi* MiNT database contains almost 14% of the nuclear genes. Following the same approach, similar results were obtained for the two other trypanosomatid models *T*. *brucei* and *L*. *major*, that together with *T*. *cruzi* conform the so called TriTryps. The presence of peptide signals was studied and a conserved peptide signal, M(L/F) R (R/S) SS, named TryM-TaPe, was found in 60% of the genes in the database. In addition, several nucleotide conserved elements were detected in both 3’ and 5’ untranslated regions. Amongst them, a compositional conserved element, DARRVSG was identified. Considering that in *T*. *brucei* this element is recognized by the TRRM3 protein which is mainly associated to the mitochondrial membrane [23], we here suggest a putative zip code role for this conserved element. Globally, the results here presented not only provide an inventory of the mitochondrial proteins in *T*. *cruzi*, but also give evidence for the existence of both peptide and mRNA signals specific to nuclear encoded mitochondrial proteins.

## Materials and methods

### Databases

This work was performed using data from the TriTrypDB 33 available at Tritrypdb.org [24].

### UTR sequences

To determine the boundaries and sequences of the UTRs of the transcripts in *T*. *cruzi* RNASeq data from Smircich *et al*. [25] and the UTRme software was used [26].

### Ortholog genes

TriTrypDB tools were used for massive ortholog gene finding when data for compared organisms were available. When this approach was not possible, Best Reciprocal BLAST hit was performed [27], applying in house bash scripts developed for this purpose.

### Gene ontology

Annotation of Gene ontology (GO) terms was performed using the online tool DAVID (Database for Annotation, Visualization and Integrated Discovery, v6.7) [28]. Background databases employed were different, as indicated in each case, depending on the analysis performed. This tool was also used for GO term enrichment analysis. As reported [29], an enrichment score higher than 1.3 was accepted as meaningful. A variant of the Exact Fisher Test (EASE score) was used for p-value calculation. For each ontological category the FDR (false discovery rate) was controlled with the Benjamini method [30], considering acceptable only those values lower than 0.05. Alternatively, TriTrypDB tools were used to analyze GO term enrichment of the gene lists using the whole genome as background. In this case the p-value cutoff was set on 0.01 and the search was limited to GO Slim terms.

### Compositional and structural analysis

Both CDS and UTR GC content was computed with Geecee tool (EMBOSS package). On the other hand, GC content at third codon position (GC3) for CDS was estimated using the INCA software (INteractive Codon usage Analysis) [31]. This tool also allowed the analysis of the codon usage bias using the MELP measure to quantify synonymous codon usage (MILC: Measure Independent of Length and Composition; MELP: MILC-based Expression Level Predictor) [32]. The RNAfold algorithm (Vienna RNA v2.0 package) was used to infer the minimum free energy (MFE) structure and the thermodynamic stability of the whole or partial region of the transcripts [33]. MFE was computed separately for the untranslated regions or the coding sequence.

### Conserved sequence signals

The search for sequence conserved signals was done using the MEME suite package tools [34]. The elicitation of discriminative regular expression motifs in specific data subsets was performed using DREME tool [35], applying the gene complement of the query subset as negative control. The motifs retrieved were compared against Ray motifs’ database, using TOMTOM tool [34] from the same package. MEME tool was used to generate consensus regular expressions when comparing motifs from different searches.

When studying signal peptides, three different approximations were made. The first approximation was performed using MEME and FIMO tools [36] both from MEME suite package with a threshold = 0.001. In addition, TargetP [16] and PredSL [37] signal peptide predictors were used with the default parameters selecting non-plant sequence.

For the motif searching analysis we chose to use a 5’ and 3’UTR length corresponding to the 60^th^ percentile of the distribution (S1 Table). For the 3’UTR, this gives 350 nt (3’UTR_350_), while for the 5’UTR the length corresponds to 100 nt. With the purpose of comparing and controlling results obtained for both UTRs it is desirable to achieve equal length for both UTRs, so the 5’UTR was defined as the region starting at -100nt from the AUG to +250 nt downstream from it (5’UTR_350_).

## Results and discussion

### Construction of MiNT: A database of nuclear genes encoding mitochondrial proteins in *T*. *cruzi*

**Fig 1:**
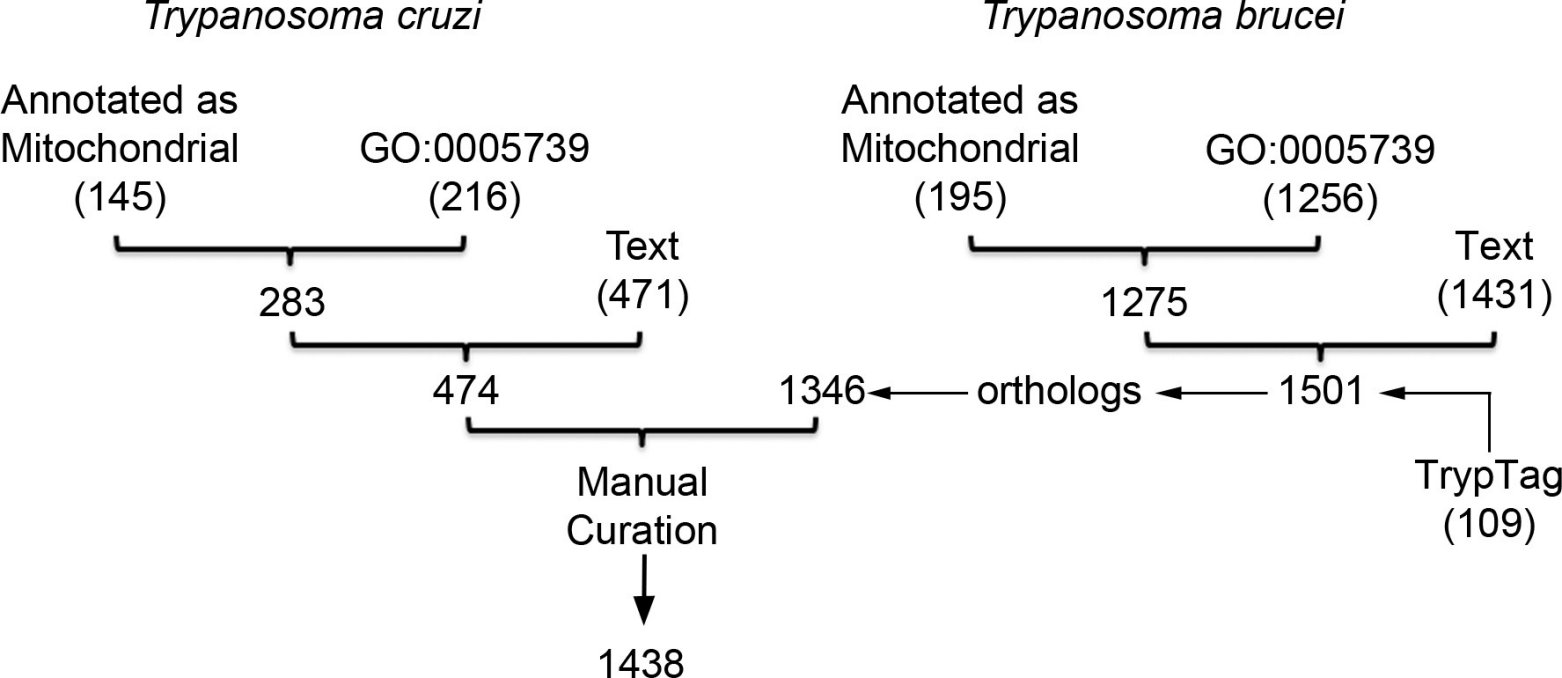
Schematic representation of the *in-silico* strategy to build a database of the nuclear genes encoding mitochondrial proteins in *T*. *cruzi*. The genomes of the mentioned trypanosomes were interrogated under selected categories (annotated as mitochondrial, GO and Text). For each, the output number of genes is shown underneath in brackets. The braces point to the number of genes obtained by the union of the involved outputs. When performed on the *T*. *cruzi* genome, the search yields only 474 genes. The same strategy in *T*. *brucei* genome, due to the state of the annotation, allowed to define a database containing 1501 nuclear genes encoding mitochondrial proteins. By comparing this later with the *T*. *cruzi* genome, a database of 1438 nuclear genes encoding mitochondrial proteins could be obtained.

In order to generate a database that comprehensively contains the nuclear transcripts that encode mitochondrial proteins (MiNT) in *T*. *cruzi*, an inclusive strategy was followed. First, all the genes whose products were annotated as “mitochondrial” in TriTrypDB 33 were used to conform a base-set which, surprisingly, consisted only on 145 genes. As this strategy revealed meager results, several other complementary approaches were performed (Fig 1). Using TriTrypDB tools, a search by ontology terms (Cellular component = "Mitochondrion"; GO:0005739) was done, obtaining 216 genes. As expected, most of these genes were included in the initial data-set, yielding only a little increment (total 283 genes). Therefore, to further expand the *T*. *cruzi* database, we use the TriTrypDB search tool based now on text, which allows the exploration of the enclosing author notes. We reasoned that this search could help to detect genes encoding mitochondrial products that have not yet been updated in regard with their function, localization or even for their complete sequence. Using this strategy and after a manual revision of the results, 474 genes including 193 new and 281 already present in the database, were identified. The inclusion of these new genes is supported by the enriched terms associated to cell respiration and Krebs cycle, found for this subset through Functional Annotation Clustering tool (DAVID). Since, in the case of *T*. *brucei*, at least 1065 mitochondrial proteins encoded by the nuclear genome were predicted [15], we considered that the current number of genes in the MiNT database was still too low.

Following the above described search approaches in *T*. *brucei*, we identified 195 genes annotated as mitochondrial products, 1256 genes under the GO term (GO:005739) and 1431 genes based on text search, summing up a total of 1501, out of the 11703 nuclear genes (13%), encoding mitochondrial products (S2 Table). This database includes not only all the genes that encode mitochondrial products as observed by fluorescent protein tag (109) [38] and most of the genes (1049/1061) proposed by Xiaobai Zhang *et al*. [15] but also adds 452 more genes. It was not surprising to found that our strategy in *T*. *brucei* yielded a higher number of nuclear genes encoding mitochondrial products than in *T*. *cruzi* since the annotation of the *T*. *brucei* genome is much more complete than the one of *T*. *cruzi*.

As a final approach, we decided to search for the *T*. *cruzi* genes orthologs to the ones we identified in *T*. *brucei* following our strategy. After manual revision, 1346 genes were found to have an ortholog gene in *T*. *cruzi*. With this strategy, 994 new genes were added to the *T*. *cruzi* database. Thus, we completed the database named as MiNT which, after a final manual curation, contains a total of 14% nuclear genes (1438 out of 10597) encoding mitochondrial proteins (S3 Table).

The same strategy was also performed to define the mitochondrial proteins encoded in the nuclear genome in *L*. *major*. As in *T*. *cruzi*, the results of the search by annotation, GO:0005739 or text (175, 313 and 467 genes respectively) gave a poor number of only 477 genes. As before, we added to this group *L*. *major* orthologs to *T*. *brucei* and *T*. *cruzi* MiNT genes. From *T*. *brucei* MiNT, 1433 ortholog genes in *L*. *major* were identified, increasing the number of nuclear genes encoding mitochondrial proteins to 1490. In addition, when using *T*. *cruzi* MiNT 1271 orthologs in *L*. *major* were found, yielding a MiNT database of 1558 gene (S1 Fig and S4 Table).

As expected, considering the number of genes encoding hypothetical proteins in *T*. *cruzi*, a great number of the MiNT genes (36%, 519/1438) are annotated as such. Nonetheless, 97% of them (501/519) have an ortholog gene either in *T*. *brucei* or in *L*. *major*, strongly suggesting a shared functional role in these organisms.

In brief, following a strict inclusion strategy to avoid false positives, we found that around 15% of the TriTryps’ nuclear genome encodes mitochondrial proteins (14%, 13% and 17% for *T*. *cruzi*, *T*. *brucei* and *L*. *major* respectively).

### Compositional and structural analysis of *T*. *cruzi* MiNT

In order to study the compositional and structural features of the nuclear genes encoding mitochondrial products in *T*. *cruzi*, the transcripts belonging to MiNT were split into the CDS and UTRs and both were independently analyzed.

**Fig 2:**
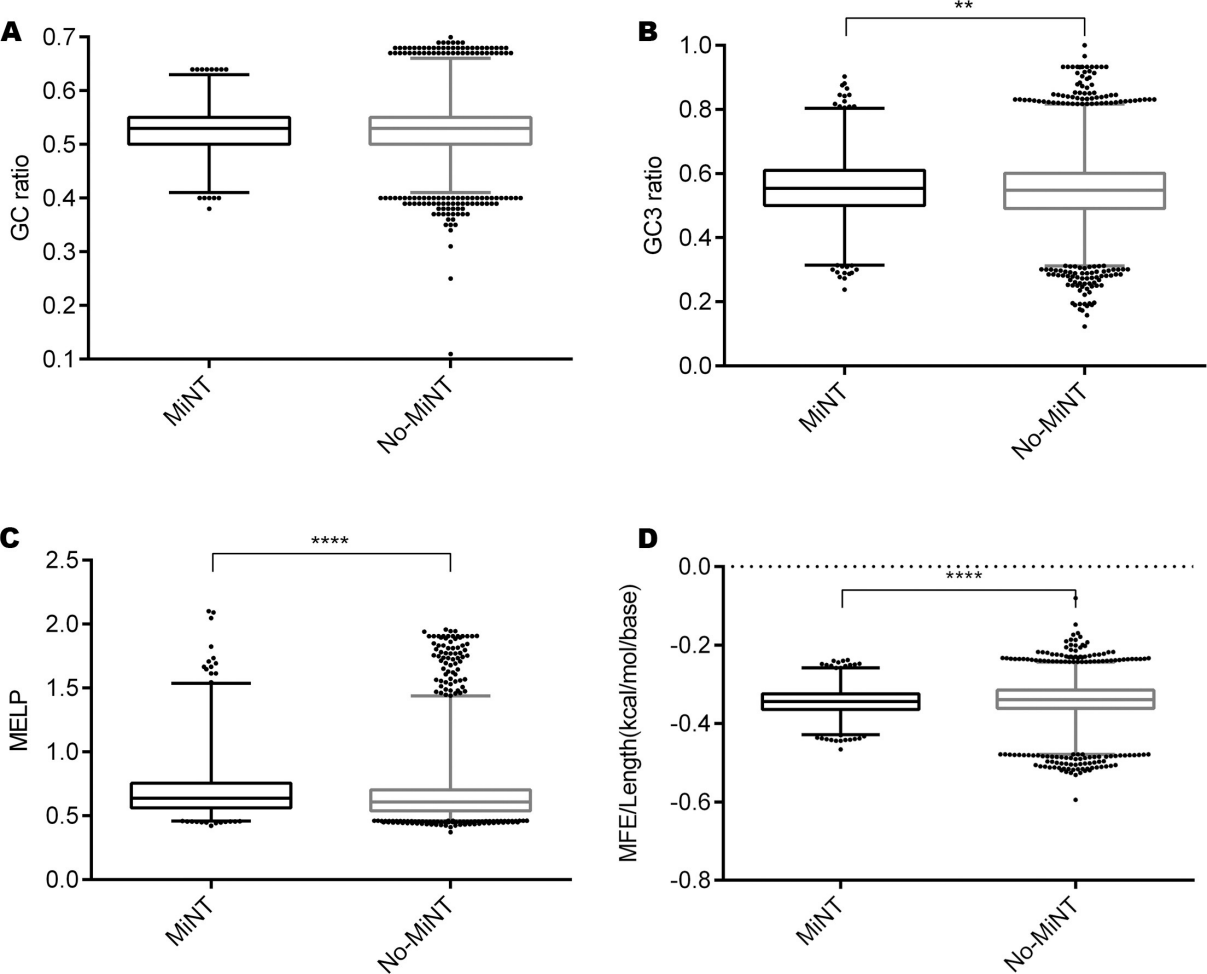
Analysis of MiNT vs No-MiNT CDS parameters. Box-plot with whiskers (percentile 1–99%) of (A) G+C Content ratio (*p-value* 0.4321), (B) G+C in the third codon position ratio (p-value < 0.002), (C) MELP, Measure Independent of Length and Composition -based Expression Level Predictor, (*p-value* < 0.001) and (D) MFE (minimum free energy)/Length ratio (*p-value* < 0.001).

For the CDSs, no significant differences were found when comparing length (S2 Fig) or GC content (Fig 2A). However, both GC3 and the MELP (Fig 2B and 2C) were significantly higher in MiNT transcripts than in the rest of the transcriptome suggesting high protein production. Another difference between MiNT CDSs and the CDSs of the rest of the transcriptome (No-MiNT) is found at the structural level. Indeed, in accordance with the mitochondrial prokaryotic origin [39], lower structured CDS are predicted by the MFE per base analysis (Fig 2D) for the nuclear derived transcripts encoding mitochondrial proteins.

**Fig 3:**
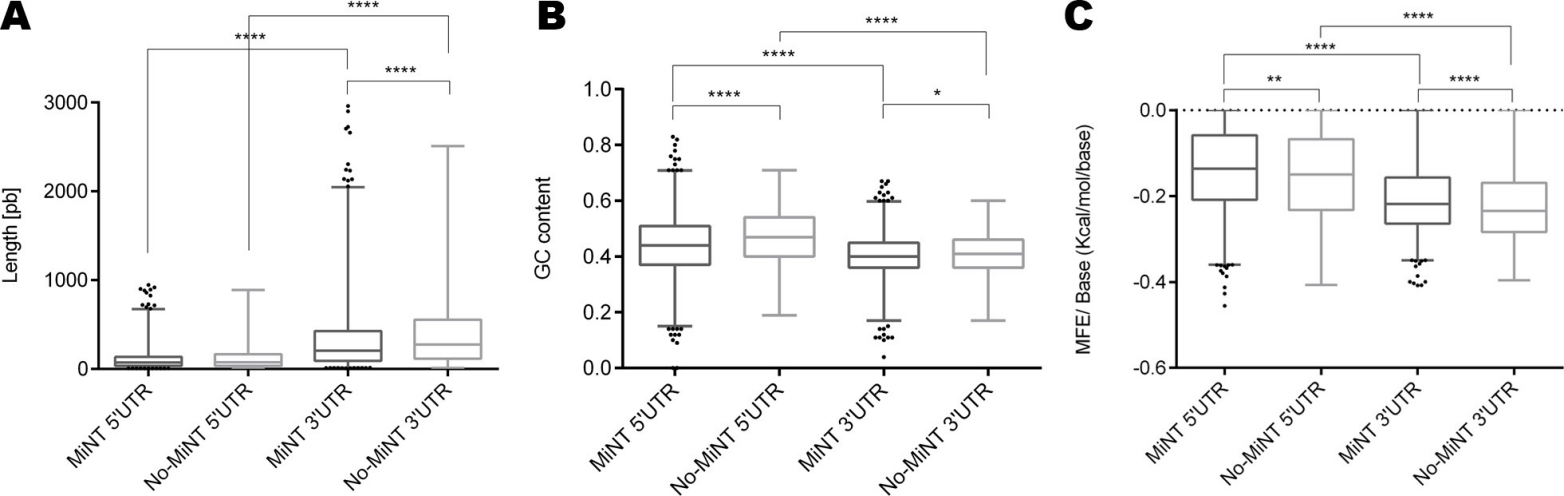
Analysis of MiNT vs No-MiNT UTRs parameters in epimastigote stage. Box-plot with whiskers (percentile 1–99%) of (A) Length; (B) G+C Content ratio (C) MFE (minimum free energy)/Length ratio. Multiple comparisons amongst groups were performed by Dunn’s multiple comparison test and differences were seen by comparing mean ranks.

We also performed a comparison among MiNT and No-MiNT UTRs in epimastigotes (Fig 3). While no differences were observed when comparing the length of the 5’UTRs, MiNT 3’UTR are significantly shorter than those of No-MiNT genes (Fig 3A). Regarding the GC content, both 5’ and 3’ UTR of MiNT present a lower GC content than the ones of No-MiNT genes (Fig 3B). Finally, considering the minimum free energy level, the predicted secondary structure for both 5’ and 3’UTR were found to be less stable in MiNT than in No-MiNT (Fig 3C). Similar results were obtained using the UTRs from the metacyclic trypomastigote stage (S3 Fig)

The distinctiveness observed for the coding and not for the regulatory regions of the nuclear genes encoding mitochondrial proteins may be explained by the more stringent requirements that govern their functionality. Indeed, the codon usage is highly non-random with respect to both GC3 and MELP.

Overall, the compositional and structural analysis of the nuclear encoded mitochondrial genes of *T*. *cruzi* revealed both high expression characteristic values (higher GC3 and MELP) and prokaryotic origin traces (less structure complexity at CDSs and UTRs) when compared with no-MiNT.

### Expression analysis of MiNT genes along the life cycle of *T*. *cruzi*

Expression evidence for the 99% of MiNT genes (1427/1438) whether in micro-arrays data [40], expressed sequence tags (EST) or RNA-seq data has been reported [25,41].

**Fig 4:**
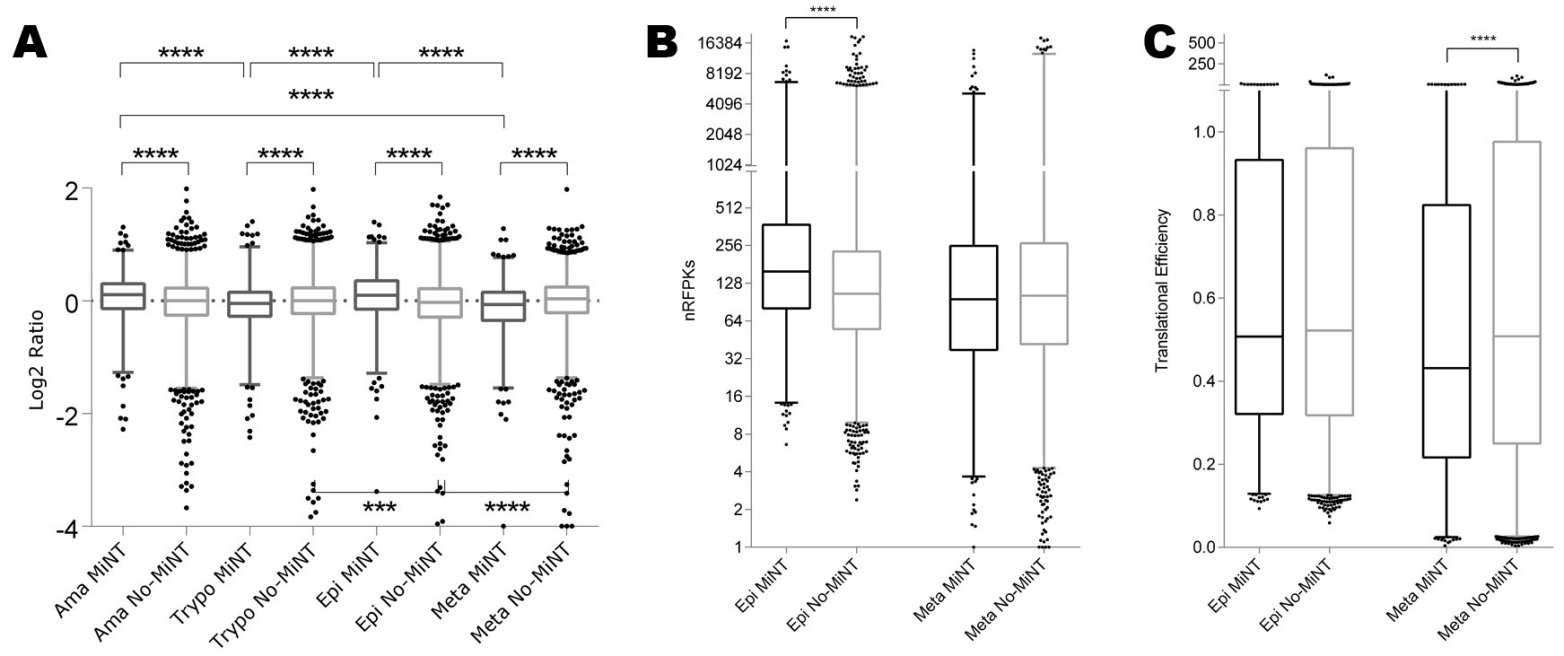
Comparison of expression data for MiNT and No-MiNT genes in *T*. *cruzi* life cycle. (A) Transcriptome data obtained from the microarray approach of Minning, et al. [40] as a ratio of the particular stage to an equal mixture of all four life cycle stages. (B) Transcriptome data obtained from RNA-Seq from Smircich, *et al*. [25] as number of reads per kilobase nRPK. (C) Ribosome footprinting data from RNA-Seq from Smircich, *et al*. [25] as number of footprints per transcript (translational efficiency). Parasite stages: Ama = amastigote; Trypo = trypomastigote; Epi = epimastigote; Meta = Metacyclic.

First, the micro-arrays data published by Mining *et al*. [40], was used to compare MiNT gene expression across the life cycle of the parasite (Fig 4A). This analysis revealed that MiNT genes have an exacerbated expression when compared with the rest of the genome, being higher in the replicative stages, and lower in both trypomastigotes forms. Similar results were obtained using the RNA-Seq data from Smircich *et al*. [25] (Fig 4B).

As a complementary approach, the analysis of ribosome footprinting data available for the vector parasite stages (epimastigote / metacyclic trypomastigote) [25] was performed (Fig 4C). Higher ribosome occupancy for MiNT when compared to the rest of the genes at the replicative epimastigotes was revealed. No such effect was observed at the metacyclic trypomastigote stage. Indeed, the translation slowdown that is a characteristic of this infective stage is also clearly observed for MiNT.

### Search for an amino-terminal localization peptide signal in mitochondrial proteins encoded by nuclear genes in *T*. *cruzi*

While the presence of a signal peptide targeting proteins to its final localization is not mandatory, at least 70% of the mitochondrial proteins are demonstrated to carry a peptide that is responsible for their subcellular localization in yeast [42]. Certain loosely characteristics, such as an amphipathic character and the presence of at least two basic amino acids have been proposed for the mitochondrial signal peptide [43]. Nonetheless, there is not a conserved consensus sequence reported for this signal.

**Fig 5:**
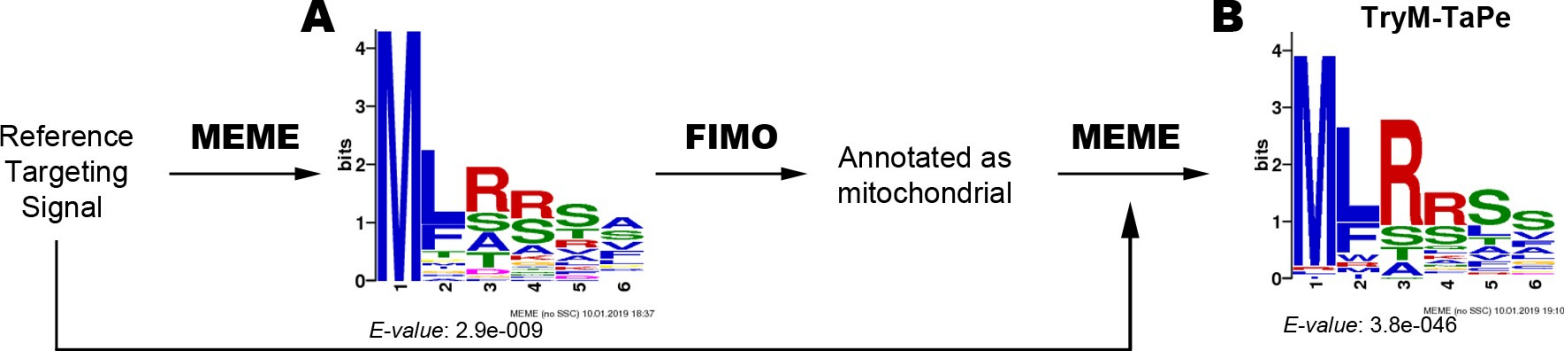
TryM-TaPe definition strategy. Mitochondrial targeting sequences available in [44] were used to obtain a preliminary signal (A) that was then used to search among the mitochondrial annotated proteins. This later search output was used as an input to obtain the final signal named as TryM-TaPe (B).

Aiming to define a consensus sequence to identify those proteins whose localization could be directed by a signal encoded in the aminoacidic sequence in *T*. *cruzi*, we firstly analyzed several experimentally tested signal peptides. Thirty-five previously reported signals [44] were used as an input to define a preliminary consensus sequence (Fig 5A). It was then submitted to FIMO analysis using the mitochondrial annotated proteins as the target database. MEME analysis found a consensus mitochondrial targeting sequence named as TryM-TaPe (Trypanosomal Mitochondrial Targeting Peptide) (Fig 5B).

We extended the search of TryM-TaPe to the complete MiNT database and found that 865 out of 1438 encoded proteins of MiNT database (60%) contained this conserved sequence, a number that is consistent with reports in other eukaryotes [42]. Though we cannot rule out the presence of other peptide signals, the existence and representation of TryM-TaPe validates the reliability of the *T*. *cruzi* MiNT database.

### Search for mRNA localization signals in nuclear genes encoding mitochondrial proteins in *T*. *cruzi*

The absence of a peptide signal in nuclear encoded mitochondrial proteins may be overcome by the presence of signals in the transcript mediating the approach of mRNAs to the mitochondria. To facilitate the search for these transcript localization signals, the identification of a reliable set of proteins without a mitochondrial localization sequence (MTS) would be advisable. For this purpose, we firstly identified all proteins in MiNT with an MTS (MiNT-MTS dataset) to then obtain those without MTS (MiNT-NoMTS dataset).

**Fig 6:**
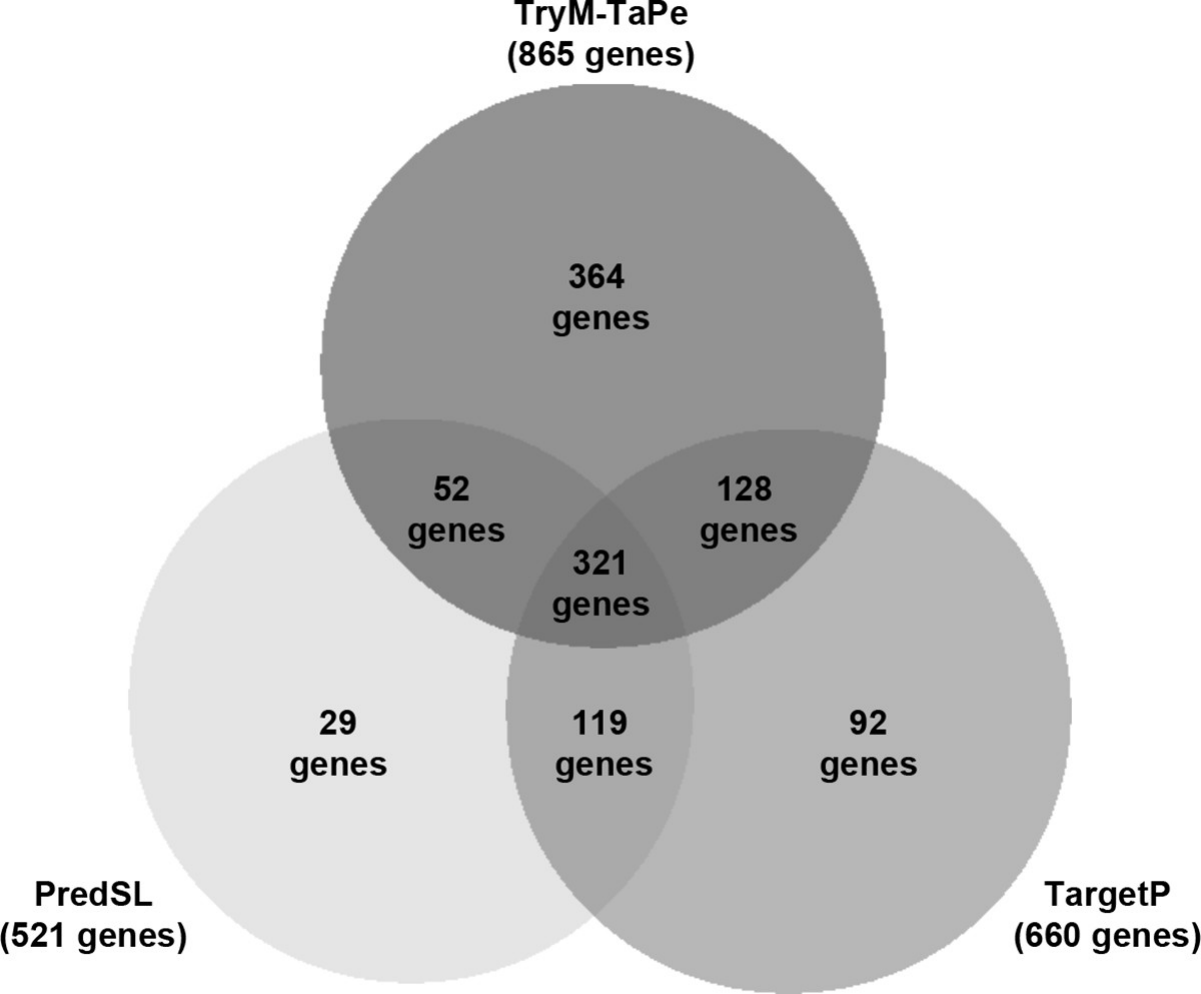
Venn diagram of the different strategies used to define the subset of nuclear encoded mitochondrial protein carrying a signal peptide in *T*. *cruzi*. The signal peptide predictors TargetP [16] and PredSL [37] predictors with the default parameters selecting non-plant sequence, and FIMO using TryM-TaPe were used on the *T*. *cruzi* MiNT database.

Since TryM-TaPe may not be the only signal peptide that could be acting as a mediator for transcript or protein localization to the mitochondrial surrounding, two common MTS predictors (TargetP and PredSL) were also used. As shown above, FIMO search led to 865 proteins carrying TryM-TaPe, meanwhile TargetP and PredSL predicted 660 and 521 proteins encoded by MiNT transcripts carry an MTS, respectively (Fig 6). We decided to include in MiNT-MTS those genes predicted by at least two of the three methods (620), while the remaining 818 genes correspond to MiNT-NoMTS.

In order to investigate the presence of sequence elements enriched in MiNT-NoMTS with respect to MiNT-MTS, we analyzed the UTRs of those transcripts. Considering that nucleotide motifs may also complement the function of the signal peptide, the reciprocal search was also carried out. We used the DREME tool [35] on the UTR_350_ (as described in Materials and Methods) to search for enriched motifs within each database and the TOMTOM tool [45] to search for putative interacting RBPs. As the database used in TOMTOM [46] includes *L*. *major* and *T*. *brucei gambiense* proteins, *T*. *cruzi* orthologs were searched.

The analysis of MiNT-NoMTS yielded 21 and 20 motifs in the 5’UTR_350_ and 3’UTR_350_ respectively and several interacting proteins were predicted (S5 Table and Table 1). Common trans-acting factors such as: the protein PABP (polyadenylate-binding protein), a general factor implied in different steps of mRNA metabolism and the protein DRBD12, known to destabilize the wide spread ARE-containing target genes[47], were found to interact to motifs in both the 3’UTR_350_ and 5’UTR_350_. In addition, motifs recognized by DRBD3 (one) and TRRM3 (three) were also found. In *T*. *brucei* DRBD3 was found to be associated with mRNAs encoding membrane proteins, playing a role in mRNA stabilization, splicing, translation and transport [48]. Interestingly, TRRM3 is found within the mitochondrion or associated to its membrane in *T*. *brucei* procyclic forms [23]. As usually found in mitochondrial proteins, TRRM3 is regulated by arginine methylation [49]. Therefore, it is tempting to propose a zip code role for the TRRM3 target motifs.

**Table 1:** Enriched motifs found in MiNT-NoMTS database.

MiNT-NoMTS vs No-MiNT						
**5'UTR**						
**Motif **	**Consensus Sequence**	**P-value **	**TomTom Result **	**Product Description**	**Ortholog gene**	**Product Description**
Motif 1	MAAAR	4.8e-008	Tbg972.9.5210	polyadenylate-binding protein 1	TcCLB.506885.70	polyadenylate-binding protein 1, putative
			LmjF.35.4130	polyadenylate-binding protein 2	TcCLB.506885.70	polyadenylate-binding protein 1, putative
Motif 2	TTGTKATT	4.0e-009	Tbg972.7.6230	Double RNA binding domain protein 12	TcCLB.506825.10	Double RNA binding domain protein 12
Motif 3	TAGAATGG	2.6e-008	Tbg972.3.3970	Triple RNA binding domain protein 3	TcCLB.510149.140	Triple RNA binding domain protein 3
Motif 4	AGAGAGGT	9.3e-010	Tbg972.6.2300	RNA-binding protein, putative	TcCLB.509965.180	RNA-binding protein, putative
**3'UTR**						
**Motif **	**Consensus Sequence**	**P-value **	**TomTom Result **	**Product Description**	**Ortholog gene**	**Product Description**
Motif 5	AAMARA	2.5e-020	Tbg972.9.5210	polyadenylate-binding protein 1	TcCLB.506885.70	polyadenylate-binding protein 1, putative
			LmjF.35.4130	polyadenylate-binding protein 2	TcCLB.506885.70	polyadenylate-binding protein 1, putative
Motif 6	GDAAA	5.8e-014	Tbg972.9.5210	polyadenylate-binding protein 1	TcCLB.506885.70	polyadenylate-binding protein 1, putative
Motif 7	TTGTYGTT	2.4e-008	Tbg972.6.2300	RNA-binding protein, putative	TcCLB.509965.180	RNA-binding protein, putative
Motif 8	HGTAG	5.5e-008	Tbg972.11.17950	RNA-binding protein, putative	TcCLB.507037.20	RNA-binding protein, putative
Motif 9	AGAW	1.1e-012	Tbg972.11.17950	RNA-binding protein, putative	TcCLB.507037.20	RNA-binding protein, putative
Motif 10	TTMTTW	7.2e-011	Tbg972.9.4840	Double RNA binding domain protein 3	TcCLB.506649.80	Double RNA binding domain protein 3
			Tbg972.7.6230	Double RNA binding domain protein 12	TcCLB.506825.10	Double RNA binding domain protein 12
Motif 11	ARRGGG	2.0e-019	Tbg972.3.3970	Triple RNA binding domain protein 3	TcCLB.510149.140	Triple RNA binding domain protein 3
Motif 12	WAGG	3.8e-018	Tbg972.3.3970	Triple RNA binding domain protein 3	TcCLB.510149.140	Triple RNA binding domain protein 3
Motif 13	GAAGCCC	9.8e-009	Tbg972.3.3970	Triple RNA binding domain protein 3	TcCLB.510149.140	Triple RNA binding domain protein 3

For MiNT-MTS we found 12 overrepresented sequences in the 5´UTR_350_ and 12 in the 3´UTR_350_ (S6 Table). As expected, and validating our approach, the sequence encoding TryM-TaPe was found. TOMTOM analysis allowed to associate four motifs of the 3’UTR_350_ to RNA binding proteins (Table 2). Two of them are recognized by the general factor PABP and two others by two isoforms of the double RNA binding domain, DRBD3 and DRBD9. Remarkably, one motif is recognized by TRRM3. As mentioned above, this is not surprising since subcellular localization signals may act in collaboration with peptide signals. Thus, this finding reinforces the proposed role. All the sequences, in the 3’UTR_350,_ found to be associated to TRRM3 (Motifs 11, 12, 13 and 15) were used to obtain a consensus recognition motif (DARRVSG) which in turn, can also be recognized by TRRM3 according to the TOMTOM algorithm (*p*-value 8.10 e -03).

**Table 2:** Enriched motif found in MiNT-MTS database.

MiNT-MTS vs No-MiNT						
3'UTR						
Motif	Consensus Sequence	P-value	TomTom Result	Product Description	Ortholog gene	Product Description
Motif 14	MAAA	6.6e-008	Tbg972.9.5210	polyadenylate-binding protein 1	TcCLB.506885.70	polyadenylate-binding protein 1, putative
			LmjF.35.4130	polyadenylate-binding protein 2	TcCLB.506885.70	polyadenylate-binding protein 1, putative
Motif 15	AARRA	1.4e-015	Tbg972.9.5210	polyadenylate-binding protein 1	TcCLB.506885.70	polyadenylate-binding protein 1, putative
			LmjF.35.4130	polyadenylate-binding protein 2	TcCLB.506885.70	polyadenylate-binding protein 1, putative
			Tbg972.3.3970	Triple RNA binding domain protein 3	TcCLB.510149.140	Triple RNA binding domain protein 3
Motif 16	CTTWTT	6.7e-014	Tbg972.9.4840	Double RNA binding domain protein 3	TcCLB.506649.80	Double RNA binding domain protein 3
Motif 17	GAACGCCT	1.2e-007	LmjF.35.2550	Double RNA binding domain protein 9	TcCLB.510747.80	Double RNA binding domain protein 9

It is worth noting that the RNA binding domains for all the putative interactors here presented have a high identity to the respective *T*. *cruzi* ortholog, suggesting that they could recognize the same motifs (see alignments on S7 Table).

In spite of the fact that the relevance of the motifs found will require further study, it is tempting to propose that TRRM3 and its cognate recognition motif play a role as a zip code transporting specific mRNAs to the mitochondrial surroundings.

## Conclusions

Aiming to identify conserved signals among the nuclear genes encoding mitochondrial proteins in *T*. *cruzi*, we searched for the genes annotated as such in the TriTrypDB. Despite its availability since 2005 [50], and the many efforts to its improvement, completion and annotation from there on, we only obtained meager results. Thus, we undertook the task of obtaining a comprehensive list of nuclear genes encoding mitochondrial proteins which would not only serve as the dataset target for the aim of this work but also constitute by itself a contribution to the current state of the knowledge of *T*. *cruzi* genome. Following an in-silico strategy, a wide inventory of the nuclear genes encoding mitochondrial proteins, MiNT, in the TriTryps was obtained (1438, 1501 and 1558 for *T*. *cruzi*, *T*. *brucei* and *L*. *major* respectively). The search for enriched motifs in *T*. *cruzi* MiNT allowed the identification of a list of conserved signals. Signals involved in different metabolic steps were identified. For the well-known mitochondrial localization peptides, we could establish a consensus motifhere named TryM-TaPe, M(L/F) R (R/S) SS, present in 60% of *T*. *cruzi* MiNT database. In addition, a putative mitochondrial localization role is here proposed for the nucleic element DARRVSG that may be recognized by the conserved TRRM3 protein which is enriched in the mitochondrial membrane fraction in *T*. *brucei*. While work is in progress to analyze the role of this element, its actual interaction with TRRM3 and the function of this RBP, these findings suggest that in addition to the canonical peptide localization signal, mRNA localization could be guided to the mitochondria surroundings via zip code nucleic signals present in the UTRs in *T*. *cruzi*.

## Supporting information

S1 Fig Schematic representation of the *in-silico* strategy to build a database of the nuclear genes encoding mitochondrial proteins in *L. major*. The genomes of the mentioned trypanosomes were interrogated under selected categories (annotated as mitochondrial, GO and Text). For each, the output number of genes is shown underneath in brackets. The braces point to the number of genes obtained by the union of the involved outputs. When performed on the *L*. *major* genome, the search yields only 477 genes. This result was complemented with the orthologs of *T*. *brucei* and *T*. *cruzi*.
(TIF)

S2 Fig Analysis of MiNT vs No-MiNT CDS length. Box-plot with whiskers (percentile 1–99%) of CDS length.
(TIF)

S3 Fig Analysis of MiNT vs No-MiNT UTRs parameters in trypomastigote stage. Box-plot with whiskers (percentile 1–99%) of (A) Length; (B) G+C Content ratio (C) MFE/Length ratio. Multiple comparisons amongst groups were performed by Dunn’s multiple comparison test and differences were seen by comparing mean ranks.
(TIF)

S1 Table *T. cruzi* UTR length and statistical analysis. Length obtained for both 5’ and 3’UTRs in epimastigotes and trypomastigotes stages data from Smircich *et al*. [25] using UTRme tool [26]. The descriptive statistics for each group are also included.
(XLSX)

S2 Table *T. brucei* nuclear genes encoding mitochondrial proteins. The results of each step of the search strategy are presented.
(XLSX)

S3 Table *T. cruzi* nuclear genes encoding mitochondrial proteins. The results of each step of the search strategy are presented.
(XLSX)

S4 Table L. major nuclear genes encoding mitochondrial proteins. The results of each step of the search strategy are presented.
(XLSX)

S5 Table Enriched motifs in MiNT-NoMTS UTRs. Complete set of motifs found by DREME search for both 5’ and 3’ UTR_350_. TomTom results and the ortholog gene in *T*. *cruzi*, when found, are also described.
(XLSX)

S6 Table Enriched motifs in MiNT-MTS UTRs. Complete set of motifs found in DREME search for both 5’ and 3’ UTR_350_. TomTom results and the ortholog gene in *T*. *cruzi*, when found, are also described.
(XLSX)

S7 Table RRM domain of the interacting RBPs and *T. cruzi* ortholog alignments. The RRM domain predicted by pfam in TriTrypDB for each RBP and its putative *T*. *cruzi* ortholog were aligned to confirm the similarity.
(XLSX)
