# Supplementary figures and images for: Conserved motifs in nuclear genes encoding predicted mitochondrial proteins in Trypanosoma cruzi
Source: PLoS One. 2019 Apr 9;14(4):e0215160. doi: 10.1371/journal.pone.0215160 (PMC6456187; doi:10.1371/journal.pone.0215160)

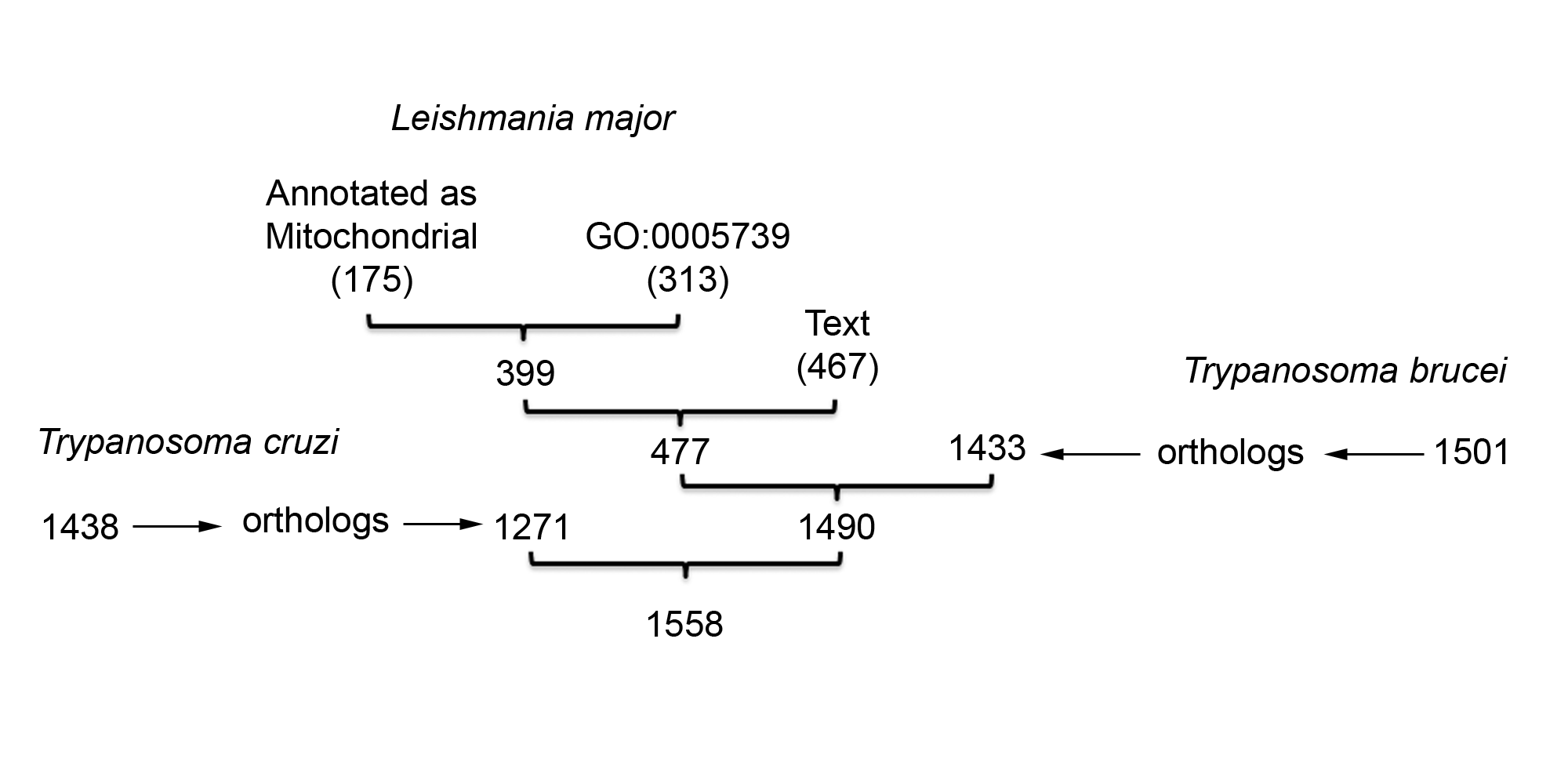

Supplement: S1 Fig — The genomes of the mentioned trypanosomes were interrogated under selected categories (annotated as mitochondrial, GO and Text). For each, the output number of genes is shown underneath in brackets. The braces point to the number of genes obtained by the union of the involved outputs. When performed on the L. major genome, the search yields only 477 genes. This result was complemented with the orthologs of T. brucei and T. cruzi. (TIF) [file pone.0215160.s001.tif]

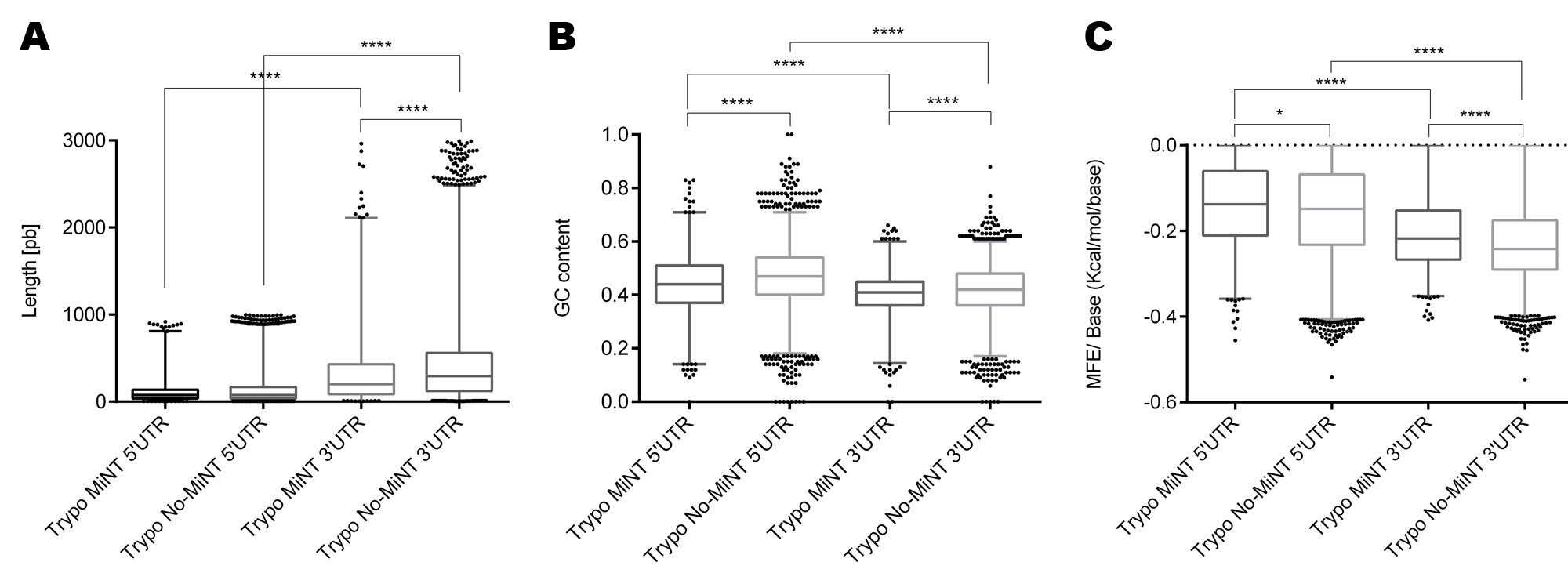

Supplement: S3 Fig — Box-plot with whiskers (percentile 1–99%) of (A) Length; (B) G+C Content ratio (C) MFE/Length ratio. Multiple comparisons amongst groups were performed by Dunn’s multiple comparison test and differences were seen by comparing mean ranks. (TIF) [file pone.0215160.s003.tif]
